# Family and social class differences in sports behavior motivation among college students: An empirical study based on the latent class model

**DOI:** 10.3389/fpsyg.2023.1070862

**Published:** 2023-01-25

**Authors:** Liping Liu, Shanping Chen, Xueyan Yang, Yuqing Yang

**Affiliations:** ^1^Department of Physical Education, Xi’an Jiaotong University, Xi’an, China; ^2^School of Public Policy and Administration, Xi’an Jiaotong University, Xi’an, China

**Keywords:** sports behavior, motivation, family social class, integration of sports and education, health, college students

## Abstract

**Objective:**

This article aims to explore the potential categories of college students’ sports behavior motivation and the differences between different family social classes on potential categories.

**Participants:**

In total, 1,092 college students were investigated in this study.

**Methods:**

This article used the “College Students’ Sports Behavior Motivation Questionnaire” to survey 1,092 college students using the whole group sampling method. The potential profile analysis method was applied to classify the types of college students’ sports behavior motivation and further analyze the characteristics of different family social classes and sports behavior motivation.

**Results:**

College students’ sports behavior motivation types can be divided into the following four categories: “low motivation type” (0.82%), “healthy appearance type” (11.45%), “healthy fun type” (37.36%), and “high motivation type” (50.37%). The higher the family’s social class, the more the sports behavior motivation of college students tends to be healthy appearance, health, fun, and high motivation. The lower the family social class, the more motivational sports behavior of college students tends to be low motivation. The better the perception of health, the higher the probability that college students belong to the high-motivation type. The fewer chronic diseases that college students have are associated with a healthy appearance and high motivation.

**Conclusion:**

There is a certain relationship between the type of college students’ sports behavior motivation and their family social class. Therefore, in school physical education, we should open all kinds of sports activities according to college students’ sports behavior motivation needs and focus on college students with lower family social class in order to intervene precisely on their motivation for sports behaviors, improve participation, and promote the overall health of college students.

## Introduction

In the new era, promoting the healthy development of youth is critical to promoting the construction of sports power. Strengthening teenagers’ fitness through exercise is significant in improving the health level of youth. In August 2020, the State General Administration of Sports and the Ministry of Education jointly issued the *Opinions on Deepening the Integration of Sports and Education to Promote the Healthy Development of Youth* (hereafter referred to as the *Opinions*). It advanced the concept of health-first education (Chinese Government Website, 2020), giving new goals and development directions to the future physical health and school sports of teenagers. However, based on the relevant studies of physical education, scholars have focused on policy formulation and goals, ignoring the intrinsic correlation between individual behaviors and policy development, psychological factors such as attitudes, and behavioral motivations of the applicable subject (Chen et al., 2017). Therefore, this study focuses on the motivation of college students’ physical exercise behavior as the entry point, analyzing and summarizing the characteristics, types, and influencing factors of current college students’ sports behavior and motivation. This provides theoretical guidance and practical insight for better promoting physical health education and sports work in schools, enhancing the implementation effectiveness of the *Opinions*.

Motivation is the internal psychological process or internal power that directs and sustains individual activities toward goals (Zhang and Guo, 2018). Individual behavioral motivation is important to understand the logic of individual action. Research on behavior motivation is more mature in the fields of sociology and political science. Many academic problems about sports behavior motivation wait to be solved. Sports behavior motivation refers to individuals meeting needs and demands through sports behaviors (Chen and Li, 2007). Sports behavior motivation is an important psychological variable. Research has been conducted on the current state of motivation, explanatory factors, and the influence of motivation on behavior, focusing on the influence of self-perception, behavioral attitudes, positive affective experiences, and sociocultural contexts on teenagers’ sports behavior motivation (Wang et al., 2021). Studies have explored the influence of demographic sociology variables such as gender, age, education level, and profession on teenagers’ sports behavior motivation (Wu et al., 2015). Attention has also been paid to the intensity of sports enjoyment among adolescents and how it differs in terms of gender and physical constitution (Chai et al., 2021). These studies enrich the research perspectives and content of teenagers’ sports behavior motivation. Still, the development and improvement of sports policies require more attention to structural characteristics at the macro level and understanding the heterogeneity of sports behavior motivation of different teenage groups. The social class theory provides the scientific theoretical tool to explore the motivation of youth sports behavior from a holistic perspective.

Social class is an important concept in analyzing the macro social structure as it captures differences in occupation, education, and income due to the social status of different groups throughout society (Kraus et al., 2012). Occupation, education, and income are common measurements of objective social class (Hu et al., 2014). Subjective social class measurement mainly interrogates the subjective cognition of individual level resulting from comparisons with others. The social class structure has changed significantly with economic development. It has become an important perspective for exploring structural differences in educational attainment, health levels, and public service satisfaction of social groups. Studies have demonstrated that the different sports’ needs and sports benefit acquirement in different social class groups are significantly different (Fang, 2012; Peng, 2012) and that people’s awareness and behavior of participating in leisure sports activities are significantly influenced by social class (Han et al., 2019). Certain scholars report that family social class has an important influence on youth physical activity (Pan, 2017; Andersen and Bakken, 2019), sports parenting style (Wang Fu, 2019), and sports expenditure (Jin and Zhao, 2018). On the whole, social class differences have been applied in sports research, from leisure activities to college students’ sports behaviors research. All have demonstrated the important influence of social class.

The impact of family social class on individual development is that possession of objective material resources and the perception of subjective social status differ, which greatly impacts individual psychology and behavior (Chen et al., 2015). It is unclear whether the influence of social class differences exists in college students’ sports behavior motivation. It is not favorable to sports behavior intervention, behavior motivation guidance, and political education for college students in different social classes in the integration of sports education. This study aims to analyze the category characteristics of college students’ sports behavior motivation through the latent profile model, explore the influence of family social class on college students’ sports behavior motivation, and provide scientific intervention strategies for the formation of motivation for positive sports behaviors among college students from different family social classes, which is conducive to the reasonable and effective implementation of the integration of sports education policy.

## Research objects and methods

### Research objects

This study used the whole group sampling method to survey 1,100 first- and second-year students in a key university in Shanxi Province. A total of 1,092 valid questionnaires were collected, with a recovery rate of 99.3%. The sample details were analyzed as follows (Table 1). In all, 369 (33.8%) were female students and 723 (66.2%) were male students. The self-weight feeling of college students is between “fat” and “normal” (2.81 ± 0.910). The description of their current health is between “medium” and “poor” (3.73 ± 0.808). The degree of chronic diseases ranged between “less” and “not at all” (4.59 ± 0.673). Regarding the distribution of college students’ family and social class, 6.32% of college students were from low-class families, 30.40% were from middle- and low-class families, 40.84% were from middle- and high-class families, and 22.44% were from high-class families. These percentages are consistent with existing studies. The percentage of students from high-income families entering key undergraduate studies is higher compared with low-income families (Chen and Wei, 2013). Students from families with medium or higher income have two-thirds of the students from middle-income families and above enter key universities (Li et al., 2016), and the motivation for sports behavior is between “more strong” and “strong” (3.19–4.53).

**TABLE 1:** Descriptive statistical results of sample characteristics (*N* = 1,092).

Variable		Percentage/Mean (Standard deviation)	Variable	Percentage/Mean (Standard deviation)
Gender (%)	Female	33.8	Health motivation	4.47 (0.652)
	Male	66.2	Appearance motivation	4.31 (0.819)
Self-weight feeling		2.81 (0.910)	Fun motivation	4.35 (0.673)
Description of health		3.73 (0.808)	Ability motivation	3.98 (0.927)
Degree of chronic diseases		4.59 (0.673)	Social motivation	4.22 (0.881)
Social class (%)	Low class	6.32	Academic motivation	4.53 (0.723)
	Middle-low class	30.40	Obedience motivation	3.35 (1.143)
	Middle-high class	40.84	Economic motivation	3.19 (1.219)
	High class	22.44	Honor motivation	3.77 (1.054)

### Measurement tools

Sports behavior motivation variables: The sports behavior motivation scale was revised based on the existing five internal motivations (health, appearance, fun, ability, and social motivations) and two external motivations (academic and obedience) scales (Chen et al., 2008) with open- and closed-ended questionnaires, using the original scale questions for internal motivation and supplementing the external motivation with economic motivation and honor motivation (Chen et al., 2021). Three new economic motivations include “I want to use sports activities to get a scholarship,” “I want to use sports activities for economic benefits,” and “I want an excellent postgraduate recommendation and communication opportunities.” Three new topics of honor motivation include “I want to beat my opponent in a game,” “I want collective success,” and “I want to receive praise from others in sports activities.” The scale has 9 dimensions, with 3 questions from each dimension for 27 questions in total. All questions were rated using the Likert 5-level measure, with options very strong, strong, more strong, a little, and none with the question scores of 5, 4, 3, 2, and 1, respectively. The alpha coefficients of each subscale ranged between 0.834 and 0.955, exceeding the acceptable standard of 0.7. The criterion validity for sports exercise behavior and each subscale score ranged from 0.138 (*p* < 0.05) to 0.230 (*p* < 0.001), indicating good reliability and validity of the topics of the sports behavior motivation measure.

Family and social class variables: College students’ family and social class variables were calculated using the variables of parents’ occupational status, years of education, and annual family income. Using Lu Xueyi’s classification of social class, the higher of the two parents’ scores is used for occupational status and years of education. The occupational status devised parents’ profession into five grades as follows: unemployed and semi-unemployed class, the agricultural worker class, the industrial worker class, the business service worker class, the self-employed class and above (including state and social manager class, manager class, private entrepreneurs class, professional class, and clerk class), with a score ranging from 1 to 5 points. The higher the score, the higher the occupational class (Lu, 2003). The author uses principal component factor analysis to extract the above three variables into one factor to obtain college students’ family and social class variables.

Personal characteristics variables (Control variables): These include three items, namely, body shape, health status, and chronic diseases. All used self-assessment questions, using the Likert 5-level measure. Body shape options were ➀ obese, ➁ fat, ➂ normal, ➃ thin, and ➄ very thin. Current health status options were ➀ excellent, ➁ good, ➂ medium, ➃ worse, and ➄ poor. Chronic disease options were ➀ very serious, ➁ more serious, ➂ average, ➃ slight, and ➄ not at all (Liu et al., 2010).

### Methods of data analysis

Latent profile analysis (LPA) is a statistical method distinguishing between types of subjects, further examining the relationship between types and other variables (Liu et al., 2020). Using this method to research college students’ sports behavior motivation makes it possible to cluster but descend the multiple sports behavior motivations of college students and analyze the variability of different sports behavior motivation types.

We first used the SPSS version 22.0 statistical analysis software to conduct a descriptive statistical analysis of sample characteristics. This was followed by Mplus version 7.0 to conduct a LPA of college students’ sports behavior motivation. Finally, the SPSS version 22.0 statistical analysis software was used to explore the relationship between college students’ sports behavior motivation types and family social class using the chi-square analysis and multiple logistic regression analysis relationships.

## Results and analysis

### Latent profile analysis of college students’ motivation for sports behavior

Based on the LPA steps, the 1–6 category model was fitted to each of the nine types of sports behavior motivation of college students (Table 2).

**TABLE 2:** Six latent structure model fit indices of college students’ sports behavior motivation (*N* = 1,092).

C	LOG (L)increased	AIC reduced	BIC reduced	ABIC reduced	ENTROPY	LMRLR	BLRT
1	−12668.396	25372.791	25462.715	25405.543			
2	−11216.960	22489.920	22629.801	22540.867	0.887	**	***
3	−10641.782	21359.564	21549.403	21428.706	0.890	*	***
4	−10347.651	20791.302	21031.099	20878.640	0.916	***	***
5	−10207.297	20530.594	20820.348	20636.127	0.840	***	***
6	−10070.806	20277.612	20617.324	20401.341	0.860	0.269	***

*p < 0.1, **p < 0.05, and ***p < 0.01.

A latent profile analysis allows the homogeneous grouping of continuous data, and groups with similar data characteristics are classified into uniform subgroups (Kongsted and Nielsen, 2017). The test indexes of the model are mainly LOG/L (likelihood ratio test index), AIC and BIC (information evaluation index), and ABIC (sample correction). The smaller values of the above indexes indicate a better fit for the model. Certain scholars point out that BIC is the best among these three indexes (Burnham and Anderson, 2004). The ENTROPY index was used to assess the quality of the model classification. When ENTROPY is 0.6, it indicates that about 20% of the individuals have classification errors. When ENTROPY is 0.8, it indicates that the classification accuracy is more than 90%. The value of ENTROPY ranges from 0 to 1, and the closer ENTROPY is to 1, the better the model quality. In addition, two likelihood ratio tests, LMR and BLRT, were used to compare the fit differences of latent profile models and if the *p*-values of these two values reached a significant level (*p* < 0.05). This indicates that the model with k categories is significantly better than the model with k-1 categories (Jung and Wickrama, 2008; Rosato and Baer, 2012). Thus, among these six models, the 6-category model has the smallest BIC value, indicating that it is optimal. In terms of LMRLR, the category 2 model outperforms the category 1 model, the category 3 model outperforms the category 2 model, the category 4 model outperforms the category 3 model, and the category 5 model outperforms the category 4 model. Considering the above information, the class 4 model is better.

**FIGURE 1:**
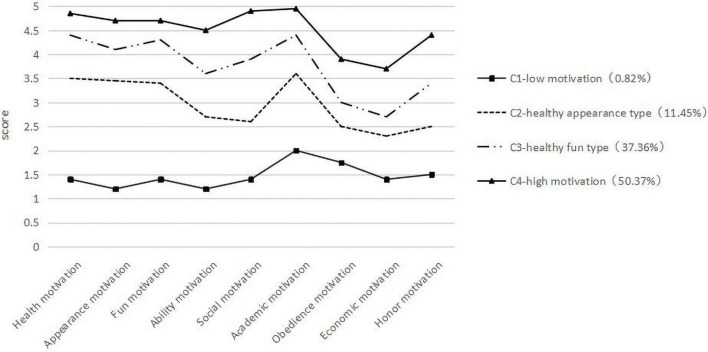
College students’ scores in nine categories of behavior motivation.

Four types of analytical models were finally selected as the final model (Figure 1), and the mean scores of the nine sports behavior motivations were an important basis for type description and naming. The bottom type of sports behavior motivation in Figure 1 has low mean scores, except for academic motivation. This is named “C1-low motivation” and has obvious differences from the other 3 types. The first five items (health, appearance, fun, ability, social) are internal motivations, and the last four items (academic, obedience, economic, and honor) are external motivations. The conditional probability of internal motivation is lower than external motivation, contrasting the three types of graphs above. Regarding the type of physical activity motivation mentioned above, except for academic motivation, the conditional probability of health and appearance motivation is high, and the conditional probability of other motivations is relatively low. Consequently, we named this type of sports behavior motivation “C2-healthy appearance type.” In addition to the high academic motivation, the probability of health and fun motivation conditions is high, and the probability of other motivation conditions is relatively low, so we named this type of sports behavior motivation as “C3-healthy fun type.” The top type of sports behavior motivation has a high probability of other motivation conditions, except academic motivation, and a relatively high probability of other motivation conditions. We named this type of sports behavior motivation “C4-high motivation” from the distribution ratio of the number of these four types of sports behavior motivation. “C1-low motivation” accounted for 0.82%, “C2-healthy appearance accounted for 11.45%, “C3-healthy fun type” accounted for 37.36%, and “C4-high motivation type” accounted for 50.37%.

### Analysis of the relationship between college students’ sports behavior motivation and family social class

**FIGURE 2:**
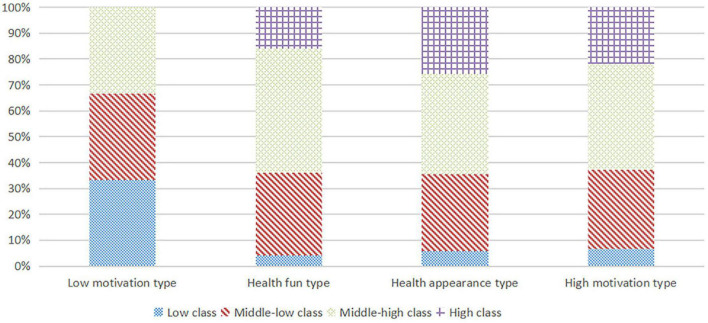
Distribution of college students’ sports behavior motivation in different family social classes.

The quartile was used to classify college students’ family social class into four categories. Those who scored 1–2 on the social class variable for university students’ families were defined as low class (69), those who scored 2–3 as middle-low class (332), those who scored 3–4 as the middle-high class (446), and those who scored 4–5 as high class (245). The cumulative percentage bar chart in Figure 2 shows the distribution of the types of college students’ sports behavior motivation among the four types of family social class. The cumulative percentage of college students’ sports behavior motivation in low-class families from high to low: low motivation type (33.3%), high motivation type (6.7%), healthy appearance type (5.9%), and healthy fun type (4.0%). The cumulative percentage of college students’ sports behavior motivation in middle-low class families from high to low was low motivation (33.3%), healthy fun (32.0%), high motivation (30.5%), and healthy appearance (29.7%). The cumulative percentage of college students’ sports behavior motivation in middle-high class families from high to low was healthy fun (48.0%), high motivation (40.9%), healthy appearance (38.7%), and low motivation type (33.3%). The cumulative percentage of college students’ sports behavior motivation in high-class families from high to low was healthy appearance type (25.7%), high motivation type (21.8%), and healthy fun type (16.0%). The chi-square test showed a significant difference in the distribution of college students’ sports behavior motivation types among the four types of family social classes (*P* < 0.05). It can be tentatively judged that the higher the family social class, the more college students’ sports behavior motivation types tend to be healthy appearance type, healthy fun type, and high motivation type. The lower the family social class, the more the college students’ sports behavior motivation tends to be low motivation type.

To further analyze the influence of family social class on college students’ sports behavior motivation, this essay constructs a multivariate logistic regression model with “college students’ sports behavior motivation types” as the dependent variable and compares the differences between healthy appearance types. For healthy fun and high motivation types with low motivation type as a reference, the family social class was the core independent variable, and gender, self-weight feeling, description of health, and degree of chronic diseases of college students were the control variables. The results in Table 3 show that the higher the family social class, the higher the probability of the healthy appearance type, healthy fun type, and high motivation type. The description of health and the degree of chronic diseases significantly affected the motivation type of college students’ sports behavior. Compared with the low motivation type, the better the description of health, the higher the probability of the high motivation type. The lower the degree of chronic diseases, the higher the possibility of healthy appearance and high motivation types.

**TABLE 3:** Multivariate logistic regression of college students’ sports behavior motivation (*N* = 1,092).

Factors of influence	Healthy appearance type/Low motivation type	Health fun type/Low motivation type	High motivation type/Low motivation type
Family social class	1.035* (0.018)	1.037* (0.017)	1.033* (0.017)
Gender	0.518 (0.840)	0.676 (0.828)	0.832 (0.827)
Self-weight feeling	1.182 (0.336)	1.159 (0.328)	1.002 (0.328)
Description of health	1.044 (0.400)	1.699 (0.391)	2.039* (0.390)
Degree of chronic diseases	1.871* (0.379)	1.809 (0.366)	2.103* (0.366)
−2 log likelihood	2052.350***		
Nagelkerke *R*^2^	0.9061		

*p < 0.1, **p < 0.05, and ***p < 0.01. Data indicate Exp (B) with SE.

## Discussion

### General characteristics of latent class of college students’ sports behavior motivation

The profile analysis in Figure 1 found that there are four potential profile types of college students’ sports behavior motivation: low motivation type, healthy appearance type, healthy fun type, and high motivation type. In addition, there are high conditional probabilities for each factor for the high motivation type and low conditional probabilities for each factor for the low motivation type. Healthy appearance type and healthy fun type are between low and high motivations. The difference between the two types mainly focuses on health, appearance, and fun. The healthy fun types are higher than the healthy appearance type. In the distribution of the four types of college students, high motivation (50.37%) > healthy fun (37.44%) > healthy appearance (11.51%) > low motivation (0.82%), “higher and lower” indicates that college students’ overall sports behavior motivation is strong. The main reasons are as follows: the increasingly rich material and spiritual culture of our times, the increased awareness of sports among students, the promotion of the diversified development of sports’ needs, and the shift of college students from passive participation to active choice in college physical education (Bai and Gao, 2022). In recent years, we have deepened the reform of physical education teaching, from the teaching content of physical education class (Hu, 2020) and course selection, forming a rich and diverse content of physical education courses conducive to improving students’ interest and motivation in physical education. It may also be related to the schools we surveyed being key universities in China. Students with exercise interests are more able to meet the actual demand for exercise at schools with higher strata. This effect is nearly half as high as that of students without exercise interests (Bai et al., 2022). Analysis of the reasons why the healthy fun type is much higher than the healthy outlook type shows two criteria. First, in China’s health policy and integration of physical education (Li et al., 2020), the new concept of physical education of “enjoying fun, enhancing physical fitness, sound personality, and refining will” (Bu et al., 2022) has greatly affected students’ sports awareness. They understand that “enjoying fun” is the foremost prerequisite for students to participate in sports. Second, it is very much in line with contemporary college students’ development needs. College students focus on appearance and pay more attention to spiritual and cultural life. This also shows that the proposed policy of integrating sports and education caters to students’ need to have fun in physical exercise.

Regarding the existential form of internal and external motivation, the high motivation type, healthy fun type, and healthy appearance type have a common ground. Their internal motivation is higher than external motivation. The low motivation type differs significantly from the other three types. The conditional probability of the first five internal motivations (health, appearance, fun, ability, and social) is lower than the last four external motivations (academic, obedience, economic, and honor). This indicates that external factors mainly influence the low motivation type of college students’ sports behavior, and their external motivation is stronger. Psychology defines internal motivation as participating in the activity for satisfaction through behavior and external motivation as participating in the activity because of external pressure, award, and rewards (Richard and Edward, 2000). From the perspective analysis of conflicting behavioral motivations, individuals intend to resist other temptations before making a behavioral decision (Atkinson and Birch, 1970; Gollwitzer et al., 2004). However, this intention to resist temptation depends on the difficulty of the motivation goal (Kruglanski et al., 2002). Internal motivation only is achieved through normal sports behavior. When students with strong external motivation cannot satisfy external needs (or find it difficult and inconvenient to finish normal behavior) according to normal sports behavior, it is highly possible to achieve through other methods of impropriety. Normally, misbehavior is a violation of the norms of sporting behavior through means other than normal sporting behavior. Sports is an important part of education. We should not only enhance the physical fitness of teenagers and promote growth and development through sports but also make students learn how to obey the rules, unity and cooperation, hard work, perseverance, and respect for others in sports to enhance the moral quality of teenagers. It is important to give full play to the value of “sports-nurturing” and “people-educating” (Liu and Li, 2020). Therefore, from the perspective of integrating sports and education, the low-motivation type groups need to be significantly concerned with physical education.

In addition, this study found that academic motivation has a higher conditional probability than other motivations among the four latent profile models. Research suggests that academic stress is the number one pressure source among contemporary college students (Wang and Yao, 2012). Due to the long-time influence of “test-based education,” students’ low health levels (Xiao and Qiu, 2020) and lack of participation and interest in sports are increasingly serious, and teenagers’ health is neglected by schools and families (Yan, 2020). Academic motivation is a double-edged sword. Moderate academic pressure may promote college students’ positive sports behavior. Nevertheless, if academic motivation is too strong, it may weaken the motivation for sports health, fun, and appearance. Students who cannot finish academic motivation through normal sports behavior will have abnormal sports behavior. It is unfavorable to form positive sports behavior and promote college students’ physical and mental health.

### Individual characteristics of latent class of college students’ physical exercise motivation

The health condition and the degree of chronic diseases have a significant influence on college students’ physical motivation types. Compared with college students of low motivation type, the better the perception of health, the greater the probability that the college students belong to the high motivation type. The less the students suffer from chronic diseases, the greater the probability that they belong to the healthy appearance type and the high motivation type. Based on the theory of exercise needs constructed by Maslow’s needs theory, exercise needs are stage-specific, directional, and selective and vary from student to student (Yu, 2019). Only when people’s basic needs, such as survival and security, are satisfied will they pursue higher-level spiritual needs As sports behavior motivation, if college students have good health without chronic diseases, they will have the most basic physical capital and the opportunity to pursue higher sports behavior motivation.

The incidence of health status and degree of chronic diseases on the type of motivation for sports behavior were compared. When comparing the low motivation type with the high motivation type, the significant effect of the health condition variable on the type of motivation [Exp (B) = 2.039, *p* < 0.1] was slightly lower than the significant effect of the chronic disease degree variable on the type of motivation [Exp (B) = 2.103, *p* < 0.1]. When comparing the low motivation type with the healthy appearance type, the chronic disease degree variable had a significant impact on the motivation type [Exp (B) = 1.871, *p* < 0.1]. Health conditions did not significantly affect the motivation type, indicating that the chronic disease degree had a greater effect on the college students’ sports behavior motivation type than a health condition. The 2019 National student physical fitness and health research results show that the problem of overweight and obesity among college students is prominent, and declining physical fitness continues (Chen and Wu, 2022). Li et al. (2019) pointed out that college students had a higher detection rate of chronic hypertension disease and better self-health evaluation among male students. Low levels of physical activity are considered an important cause of the increasing prevalence of obesity and overweight (Sultoni et al., 2017). Physical activity developed during youth reduces the probability of health-related diseases such as obesity, diabetes, anxiety, and depression, and can have lifelong health benefits (Haegele et al., 2018). It has been demonstrated that students with high-intensity physical activity have higher physical health scores than those with low physical activity (Ge et al., 2019). Motivation for physical behavior as a predictor variable of physical activity can be explained by the fact that physical health status and severity of chronic diseases also have a significant effect on motivation for physical behavior. Li et al. (2020) proposed an “integration of physical education” strategy to intervene in chronic diseases. The “integration of sports and education” needs to reflect the influence of sports, and the process of sports is the means and way of integrating sports and education. Integration can improve the physical fitness of teenagers and reduce the phenomenon of chronic diseases or obesity by means of sports.

### The influence of family social class on college students’ sports behavior motivation

This study finds that the higher the family social class, the more the college students’ sports behavior motivation tends to be healthy appearance type, healthy fun type, and high motivation type; the lower the family social class, the more the college students’ sports behavior motivation tend to be low motivation type. It has been shown that people in middle-high and high social classes with a college degree comprise the best people in civil servants, heads of enterprises and institutions, professional and technical personnel, clerks, and the commercial service industry. They have higher education, economic income, and social resources (Zhao and Hong, 2012). Their knowledge of health, sports behavior (Wang and He, 2016), and consumption behavior are higher than low- and middle-low social classes. Health is considered a responsibility and part of upbringing among the upper and middle social class. It represents the energy of life and enjoyment of life, as well as a better sense of keeping healthy, but the lower social class people see health as the ability to keep working (D’Houtaud and Field, 2010). From the analysis of the motivation of participating in leisure sports, people in high and middle-high social classes tend to pay more attention to their hobbies, sports technology of enhancement, emotional communication, enjoying life, and bodybuilding, in addition to relieving physical and mental tension by participating in leisure sports activities (Xu and Bai, 2012). People in low social class generally focus on manual labor, long work time, stressful life, and poor exercise concept and awareness (Han et al., 2019).

The family environment is an important factor influencing students’ sports behavior and motivation of behavior. Education, accompaniment, support, behaviors, and perceptions from parents implicitly influence children’s physical activity behaviors and behavior motivation. It has been demonstrated that the higher the social class of the family, the stronger the role of parental accompaniment, support, and guidance in children’s activities, and the greater possibility that children will achieve the recommended amount of physical activity. Therefore, the higher the family social class, the higher the influence of family parents, and the higher the motivation for sports behavior in terms of health, fun, and appearance; on the contrary, the lower the family social class, the lower and negative influence of parents in terms of sports behavior motivation.

### Intervention strategy of integrating college students’ sports behavior motivation

Research on the characteristics of latent class for college students’ sports behavior motivation and their relationship with family social class has important practical implications for implementing policies to promote sports and education integration.

First, from the overall characteristics of latent motivation types, some college students still belong to the low motivation type, show low interest in various sports activities, and their external motivation exceeds their internal motivation. However, the small number of this part of college students should not be ignored. Physical education of schools should pay greater attention to this group. First, through rapid, scientific, and accurate screening and evaluation, building the establishment of one-to-one teacher exchanges, support, and peer-led approaches helps students have fun, improve their health, build healthy personalities, and refine the will to comprehensively enhance their motivation to participate in physical exercise. Second, schools should strengthen the goal of “people-education” in physical education, update the physical education concept and teaching methods, and guide and encourage college students to form the habit of participating in daily physical exercise to make physical education a comprehensive role in promoting teenagers’ physical and mental health.

Second, college students with chronic diseases are still in the sports health promotion stage for disease prevention (Wang et al., 2020). In integrating sports and education, we need to treat them differently. First, the school should increase the humanistic care for them, provide free checkups regularly, combine physical with medical, and build scientific treatment and exercise prescriptions; second, offer effective physical health courses and after-school sports tutoring; and third, offer courses on prevention, treatment, and psychological counseling of chronic diseases for them.

Third, family and social class significantly influence college students’ sports behavior motivation. We should avoid the unequal sports benefits among the college student groups by improving the policy of integrating sports and education and ensuring that all college students can promote their all-round development through sports exercise. With the improvement of the family’s social class, it is evident that the pursuit of individual sports behavior for health, which has similarities with the study’s findings, poor economic condition families pay insufficient attention to the health-promoting action of physical exercise. Therefore, in the process of implementing the policy of integration of physical education, it is necessary to emphasize the development concept of promoting individual health through physical exercise, pay greater attention to low-income family students’ daily physical behaviors, and adopt appropriate support actions to help them develop the habit of exercise. Examples of this include providing them access to free stadiums and sports equipment.

## Conclusion and insights

The types of college students’ sports behavior motivation are divided into “high motivation type,” “healthy and fun type,” “healthy appearance type,” and “low motivation type.” The four types show the characteristics of “more high and less low” and a high consistency in academic motivation. In “high motivation type,” “healthy and fun type,” and “healthy appearance type,” internal motivation is higher than external motivation, and the external motivation of “low motivation type” is higher than internal motivation. The better the college students’ health condition, the more likely they are to belong to the high-motivation type. The fewer chronic diseases the students have, the more likely they are to belong to the healthy appearance and high motivation types. The severity of chronic diseases has a greater impact on the motivation of college students’ sports behavior than their health condition. The higher the family social class, the more the college students’ sports behavior motivation tends to be healthy appearance, health, fun, and highly motivated. The lower the family social class, the more the college students’ sports behavior motivation tends to be low.

This article introduces the variable of “family social class,” enriching research theory on sports behavior motivation and helping to study the influence of family social class on college students’ sports behavior and physical health from the source. We analyze the potential categories of college students’ sports behavior motivation. We also analyze the potential categories of college students’ sports behavior motivation of different family social classes to provide a theoretical basis and practical guidance for school sports management and policy formulation. This is conducive to precise intervention in college students’ sports behavior and motivation and promotes the health of all people.

There are several limitations to this study. The survey was conducted on college students in a major domestic university. Although the research results are in line with the actual situation in China, they need further research and testing on the overall and universal characteristics of college students. In the future, a series of studies might be conducted for various regions and school levels. Psychological mechanisms between willingness to regulate sports behavior, sports policy attitudes, and the types of behavioral motivation and different behavioral regulation and sports policy attitudes of students from different family social classes could also be explored.

## Data availability statement

The original contributions presented in this study are included in this article/supplementary material, further inquiries can be directed to the corresponding author.

## Author contributions

LL and SC: formal analysis and writing—original draft preparation. XY and YY: investigation, writing—review and editing, and supervision. All authors have read and agreed to the published version of the manuscript.
